# Extraction of protein from oilseed meal residues of sesame, hazelnut, almond and pumpkin produced in the oil industry for application in human and animal food

**DOI:** 10.1039/d4ra07720b

**Published:** 2025-07-16

**Authors:** Narges Rahemizadeh, Ali Gholami

**Affiliations:** a Department of Analytical Chemistry, Faculty of Chemistry, University of Kashan P.O. Box 87317-51167 Kashan Islamic Republic of Iran agholami@kashanu.ac.ir

## Abstract

In recent years, seeds and oils derived from oilseeds have become increasingly popular additives to food, so the cultivation of oil plants worldwide is growing rapidly. Alkaline extraction remains the preferred technique for protein extraction from oilseed meal in commercial processes. This study presents the current status of knowledge on the protein and amino acid composition of common oilseed meals and the nutrition, health benefits and food applications of oilseed meals containing sesame, hazelnut, almond, and pumpkin. Additionally, the amount of amino acid l-alanine in the oil seeds was determined using the ninhydrin method. The amount of l-alanine in the oilseed meal was then measured using UV-Vis spectroscopy at a wavelength of 570 nm. The percentages of protein in the isolates containing sesame, hazelnut, almond, and pumpkin were 76.20, 79.05, 81.04, and 80.22, respectively. The amount of protein in oilseed meal was determined using the Kjeldahl method for the validity test. Oilseeds are widely applied in the food industry for human and animal consumption owing to their health benefits and good functional properties.

## Abstract 2

This study presents the current knowledge on the protein and amino acid composition of common oilseed meals and the nutrition, health benefits and food applications of oilseed meals containing sesame, hazelnut, almond, and pumpkin.

## Graphical abstract

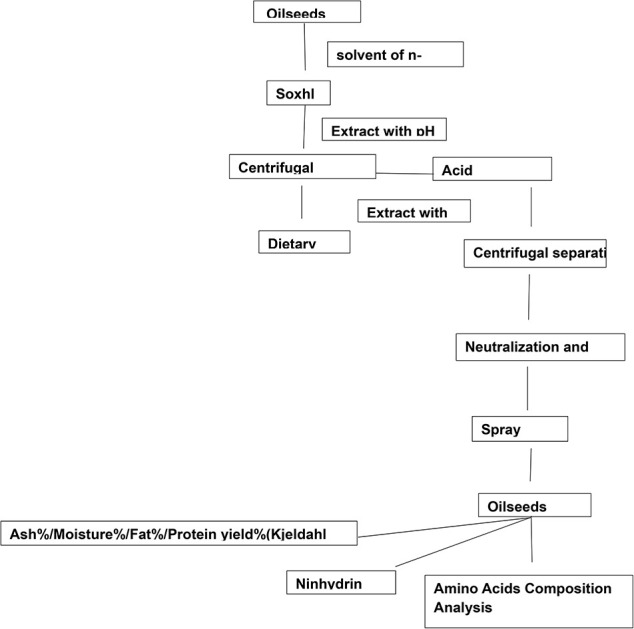

## 1. Introduction

Proteins are the most important macronutrients for humans.^1^ The global demand for protein is increasing; consequently, there is a need for new sources of dietary protein. Animal proteins are expensive in terms of market price, land requirement and environmental impact.^2^ In addition, consumer confidence in animal proteins has decreased owing to food safety concerns.^3^ The vegetable oil industry produces a lot of waste since disposal of this waste is costly and problematic from an economic and environmental viewpoint. This waste also has high nutritional and medicinal value.^4^ Therefore, waste can be used in various processes that lead to the production of high-value products and reduce the resulting production waste to a minimum.^5,6^

The animal production industry provides quality food proteins for one of the most basic human needs. This is achieved through a synergistic relationship with other segments of agriculture. The increasing global demand for protein as human and animal feed has led to increased interest in other protein sources, such as oilseed meal. The resulting meal from the oil extraction process shows promise as animal feed owing to its high crude protein content.^7,8^ The production of edible oil from oilseeds is a major sector in the global food industry. However, this prominence comes from the generation of substantial amounts of waste. The primary byproduct of the oil extraction process is oil cake. Out of the 23 million tons increase in total oil-cake production, 17 million tons are produced in developing nations, such as India, Brazil, and Argentina. Oil cake primarily comprises highly lignified husk lignocellulosic fibers (40%), proteins (35%), and phenolic compounds (5.7%).^9^

Proteins used in food processing have different origins and can be classified into animal and vegetable proteins.^10^ Oilseed meal is a good source of nutrients, such as sesame, hazelnut, almond, pumpkin and safflower seeds,^11^ especially those that contribute to human health. Many nutrients and peptides in oilseed meal are biologically active. These peptides show various activities, such as anti-blood pressure, antioxidant, anti-cancer, anti-disease, immune system modulator, and cholesterol reduction.^12^ Oilseed meal is a good source of nutritional components, especially those that contribute to human health. Many nutrients and peptides in oilseed meals are biologically active. These peptides show different activities, such as anti-hypertensive, antioxidant, anti-cancer, anti-disease, immune-modulating and cholesterol-lowering.^13^ In this research, the residue protein of sesame oilseed meal (SOM),^14,15^ protein of hazelnut oilseed meal (HOM),^16,17^ protein of almond oilseed meal (AOM),^18^ and protein of pumpkin oilseed meal (POM),^19,20^ which are rich in protein sources, were used. SOM protein is rich in methionine, cysteine and tryptophan. SOM can be added to supplements to improve the nutritional balance. SOM, an inexpensive vegetable protein source, can be used to fortify infant formula.^21^

Studies have shown that the protein of HOM can be a protein source for functional foods and, to some extent, replace existing sources in a more environmentally friendly, cheap and sustainable way. The amino acid composition of the HOM protein was comparable to that of some animal proteins and superior to that of many vegetable proteins. Additionally, the results demonstrated that hazelnut protein isolate could be an alternative and sustainable source for vegetable protein production.^22^ Almonds contain valuable sources of nutrients, such as lipids, proteins, dietary fibers, vitamins, minerals and phenolic compounds. AOM is a fat-free and high-protein meal. This meal can be valued and used as a human or animal food. Using the two-dimensional electrophoresis method, approximately 188 different proteins have been identified in almond seeds.^16,18^

Protein of POM has the highest level between 60% and 65%, which makes it an attractive and promising breeding source of plant origin. Biochemical descriptions of cultivations and functional and nutritional evaluations of POM are carried out, which can lead to the innovative use of pumpkin as a resource in different food systems as a functional element or as a complementary food.^23^

There are three ways to prepare protein isolate containing chemical, enzymatic and physical methods. One of the chemical methods is alkaline extraction, which is the most common conventional method used to extract vegetable proteins. Structural changes in an amino acid or protein that occur at different pH values change the relative solubility of the molecule. The pH value at which the concentrations of the anionic and cationic groups are equal is the isoelectric point of the protein. Amino acids and proteins have the lowest solubility at their isoelectric points.^24^ Additionally, the quantitative analysis of proteins depends on the type and nature of the protein, the nature of other components of the protein sample, and the speed, accuracy, and sensitivity of the test. Proteins contain almost the same percentage of nitrogen about 16%. The most common method for determining the amount of nitrogen in organic materials, which is based on neutralization with titration, is the Kjeldahl method.^25^ The quantitative analysis of amino acids, specific functional groups in amino acids, can react to produce specific colored products. The color intensity of the product formed by a specific group of amino acids varies according to the number of active or free groups in the reaction and their accessibility to the reagent. Methods such as ninhydrin, xanthoproteic, Hapkinsol, sodium nitroprusside, Milon, lead acetate, Pauli, and Sakaguchi have been used for the quantitative determination of amino acids.^26^

**Scheme 1:**
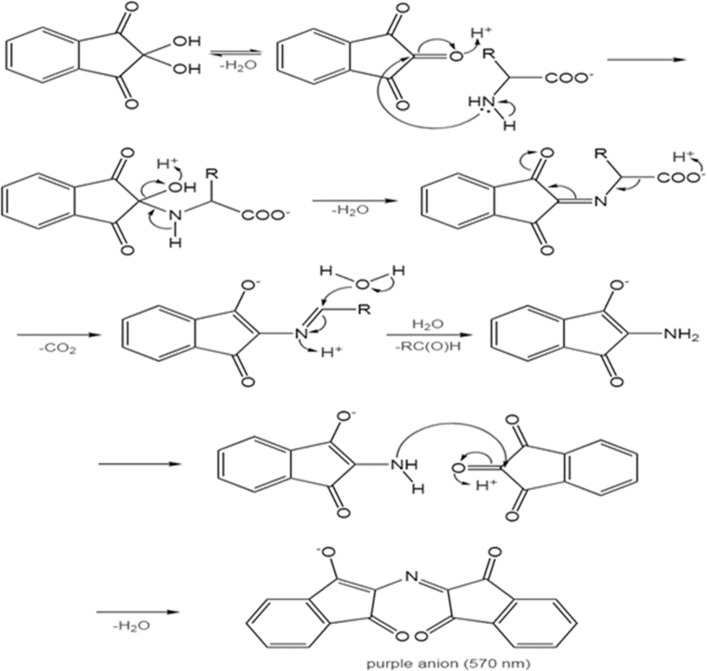
Reaction of ninhydrin with amino acids.

Amino acids consist of two parts: amines and carboxylic acids. Although instrumental techniques, such as HPLC, are currently used to determine compounds containing amino acid groups, the simple and convenient ninhydrin method still has several advantages because it does not require expensive equipment and is suitable for the routine analysis of large numbers of samples.^27^ In the ninhydrin method, amino acids pair two ninhydrin molecules through the nitrogen atom, and the amino acid is separated as an aldehyde according to Scheme 1. In this method, through the free electron pair, nitrogen reacts with the amino acid and creates a purple color. Type I and II amines respond to the ninhydrin test. According to the characteristics of amino acids, most amino acids are type 1 and type 2 amines. The absorption is checked using a spectrophotometer at a wavelength of 570 nm. One of the investigated amino acids is l-alanine. The calibration curve can be obtained using the Beer–Lambert law and the appropriate linear equation of the amino acid l-alanine and used in calculations.^26,27^

In the present study, the oilseed meal was prepared at Barij Essence Company (Kashan, Iran). The sample was produced by the cold pressing method, and it was completely crushed and powdered using a grinding machine. Then, the oil was extracted using a Soxhlet machine with an *n*-hexane solvent, and protein isolation was prepared.^28,29^ Then, using the ninhydrin test and the l-alanine absorption curve by applying the spectrophotometric method, the investigation of the quantitative measurement of sesame, hazelnut, pumpkin, and almond amino acids was performed.

## 2. Materials and methods

### 2.1. Materials

Sesame, hazelnut, almond, and pumpkin oilseed meal were obtained from Barij Essence Company, Kashan City (Iran), defatted using a Soxhlet apparatus with *n*-hexane solvent, and then turned into powder using a grinder. The sieve was passed through 20 meshes to obtain a homogeneous particle size. All the chemicals, including normal hexane, soda solution, neutral hydrochloric acid, tin(ii) chloride dehydrate, glacial acetic acid, sodium acetate trihydrate, ninhydrin, dimethyl sulfoxide, and ethanol, were obtained from Merck, Germany.

### 2.2. Measurement of some physical and chemical properties of oilseed meal

Oilseed meals should exhibit characteristics according to existing standards. In terms of appearance, they should be free of visible insects, animal droppings, pests and molds and be in the form of oilseed meal, lumps or feathers, with a good smell. The chemical composition of isolated proteins of SOM, HOM, AOM and POM was measured using the AOAC standard methods of 1990. A 105 C oven was used for moisture determination, the Soxhlet method for fat determination, the Kjeldahl method for protein determination, and a 550 C oven for ash determination.^29^ For statistical measurement, the samples were analyzed 3 times, and the measurement error was calculated.

### 2.3. Preparation of protein isolate

The protein isolate of the oilseed meal was prepared according to the method with modifications.^28^

### 2.4. Preparation of oilseed meal

An oilseed meal was prepared at Barij Essence Pharmaceutical Company. After removing the foreign substances, the oilseed meal was made into powder by a mill and passed through a sieve with 20 meshes. Then, a portion of the sample was mounted on a Soxhlet apparatus with the solvent of *n*-hexane, and oil extraction was performed for 4 hours.^30^

### 2.5. Oilseed protein extraction

**Fig. 1:**
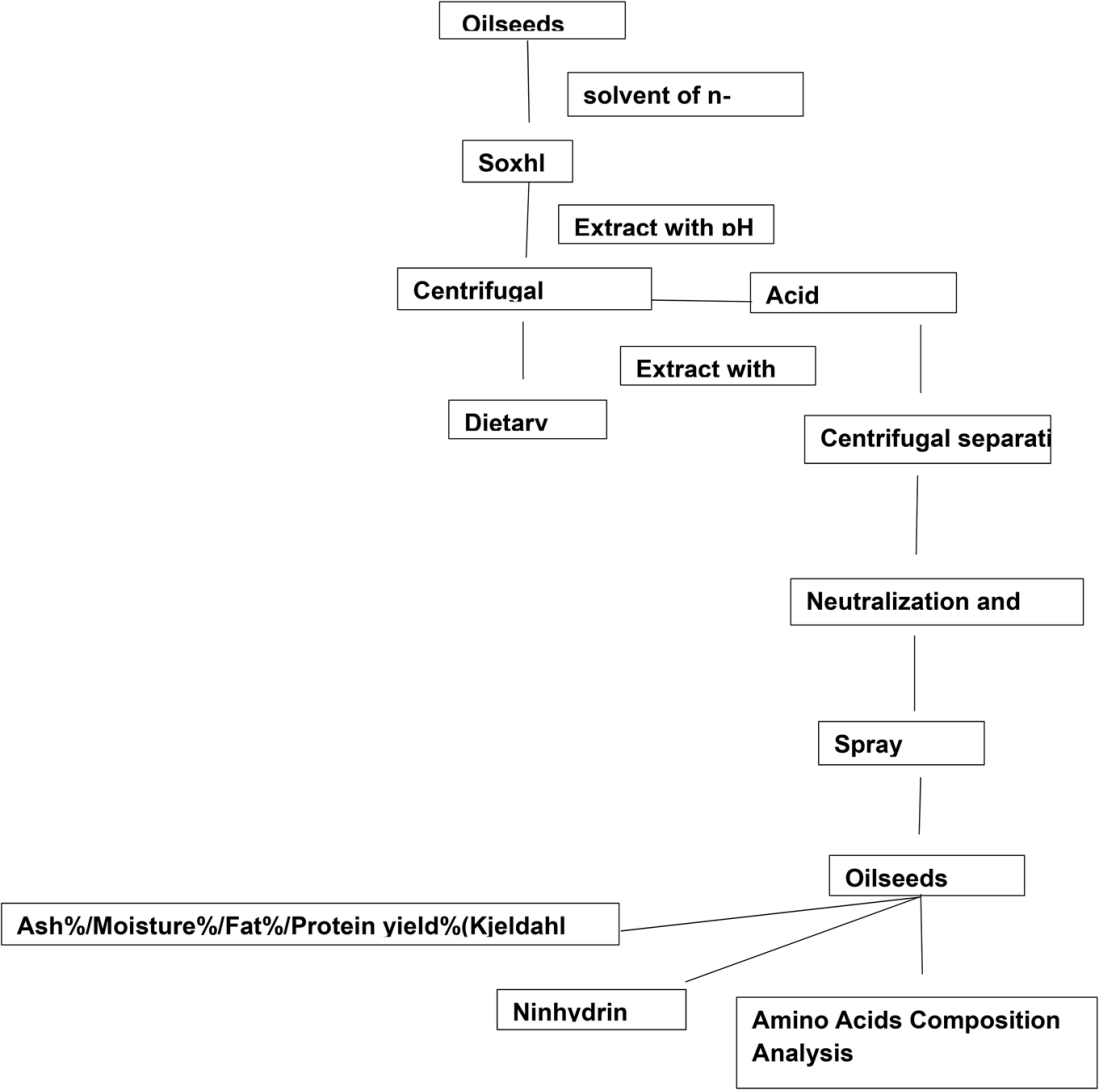
Oilseed protein extraction.

5 g of oilseed meal was degreased by *n*-hexane solvent and Soxhlet apparatus and poured into 100 mL of water; the pH was adjusted to 10.00 with soda solution and stirred with a magnetic stirrer at room temperature for 1 hour. The aqueous extract was overflowed, filtered with cotton and then centrifuged. The resulting extract was diluted with a neutral hydrochloric acid solution, and its pH was adjusted to 4.00. When the aqueous extract was neutralized, a light white sediment was separated from the extract. The remaining solution and sediment were centrifuged. The sediment separated in the test tube was washed twice with water and then dried in an oven at a temperature of 50 °C,^30^ as depicted in Fig. 1.

### 2.6. Ninhydrin test method

24 mg of tin(ii) chloride was carefully weighed with a scale (±0.0001) and then dissolved in 10 mL of sodium acetate 4.0 M. Then, 0.2 g of ninhydrin was weighed, and in 10 mL of dimethyl sulfoxide, one volume of tin(ii) chloride solution was mixed with three times the volume of ninhydrin solution to make the reagent.

A 100 mL volumetric flask was made up of volume with distilled water, and 0.5, 1.0, 2.0, 3.0, and 4.0 mL of this solution were transferred by pipette to 10 mL flasks and made up to volume with distilled water to obtain solutions with concentrations of 0.005, 0.01, 0.02, 0.03 and 0.04 (mg mL^−1^) of l-alanine should be obtained.^26,27^

### 2.7. l-Alanine amino acid calibration curve

1 mL of the newly made reagent was transferred to the Pyrex test tubes with a volumetric pipette, and 1.00 mL of each of the standard solutions was slowly added to the reagent using a volumetric pipette. The lid of the tubes was closed, and the solutions were mixed well for one minute. Then, the tubes were placed in a boiling water bath simultaneously and exactly for 15 minutes. After this time, the tubes were removed from the bath and cooled in a cold-water bath until they reached the ambient temperature. Then, with a volume pipette, 5.0 mL of 50% ethanol was added to each tube and mixed well until it was uniform. The absorbance of the solutions was read at a wavelength of 570 nm with a spectrophotometer against the control solution containing 50% ethanol.^26,27^

### 2.8. Optimization of several variables (pH–temperature–addition of salt)

To optimize the process of protein extraction from oilseed meal, the effect of pH and optimal temperature, as well as the effect of salt on increasing the yield of protein isolate, was investigated.

### 2.9. Amino acid composition analysis

The analysis of the amino acid composition of isolated protein was performed by applying the high performance liquid chromatography (HPLC) method. This instruction includes three work steps: preparation of the sample resulting from hydrolysis for chromatography, including derivatization, drying, *etc.*; isolation of the derivative obtained from the preparation stage by HPLC; and buffer for the mobile phase. Exactly 11.48 g of sodium acetate was weighed and poured into a one-liter flask. It was dissolved in 900 mL of water, and its pH was adjusted to 1.60 with acetic acid and sodium hydroxide. Then, the balloon was inflated with water. Then, 0.5 mL of triethylamine was added to this solution and mixed well. This solution was filtered with a special filter of 0.45 micrometers.

Phase A: 940 mL of the buffer made in the previous step was mixed with 60 mL of acetonitrile and degassed. Phase B: 60% acetonitrile and 40% water were mixed and degassed.^31,32^

## 3. Results and discussion

### 3.1. Physical and chemical characteristics of oilseed meal

The results related to the test of some physical and chemical characteristics of oilseed meal containing fat percentage, protein percentage, moisture percentage, and total ash percentage were calculated based on the standards mentioned in Subsection 2.2, as shown in Table 1.

**Table 1:** Physical and chemical characteristics of oilseed meal (95% confidence interval)

Oilseed meal	Ash%	Moisture%	Protein yield%	Fat%
Sesame	6.16 ± 0.21	9.60 ± 0.10	76.20 ± 0.03	12.09 ± 1.02
Hazelnut	5.12 ± 0.32	8.50 ± 0.15	79.02 ± 0.04	10.10 ± 1.03
Almonds	6.27 ± 0.2	8.40 ± 0.10	81.4 ± 0.08	11.01 ± 0.92
Pumpkin	6.24 ± 0.10	8.90 ± 0.12	80.22 ± 0.05	10.22 ± 1.01

According to Table 1, the percentage of fat extracted by the Soxhlet apparatus from all the mentioned oil seeds is in the numerical range of 10.00–12.00. Additionally, the moisture level of the oilseed meal is between 8.00 and 9.00 percent relative humidity (%RH), which is acceptable according to the standard. Moreover, the amount of ash is between 5.0% and 7.00% of ash (%ash). According to the table, pumpkin oilseed meals have the most ash. Almond has the highest amount of protein among the oilseed meals considered in this project, and pumpkin, hazelnut, and sesame are ranked next in terms of protein content.^33^

### 3.2. l-Alanine absorption calibration curve

**Fig. 2:**
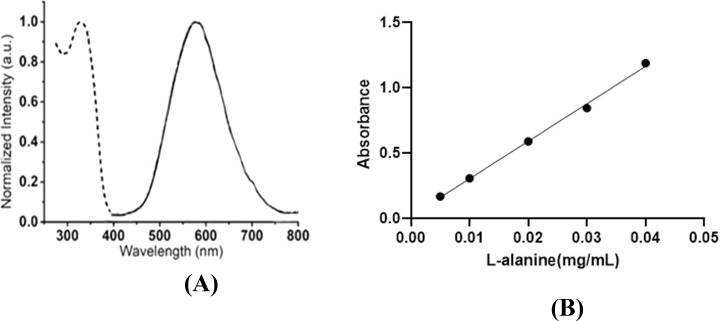
(A and B) Absorption curve according to l-alanine concentration (570 nm).

This experiment was repeated three times according to Subsubsection 2.3.4 for each concentration, and the average absorption was obtained. According to Table 2 and Fig. 2, it shows the curve of the absorption graph according to the concentration of l-alanine. Additionally, eqn (1) shows the absorption equation in terms of the l-alanine concentration.^34^

1 *A* = 28.685*C* + 0.0182** ***R*^2^ = 0.997

where *A* and *C* denote the sample absorption value and concentration of alanine solution in mg mL^−1^, respectively.

**Table 2:** Absorption according to the l-alanine concentration

*C* (mg mL)	0.005 ± 0.05	0.01 ± 0.05	0.02 ± 0.05	0.03 ± 0.05	0.04 ± 0.05
*A*	0.169	0.308	0.591	0.846	01.189

### 3.3. Optimum conditions in alkaline pH environment SOM

**Fig. 3:**
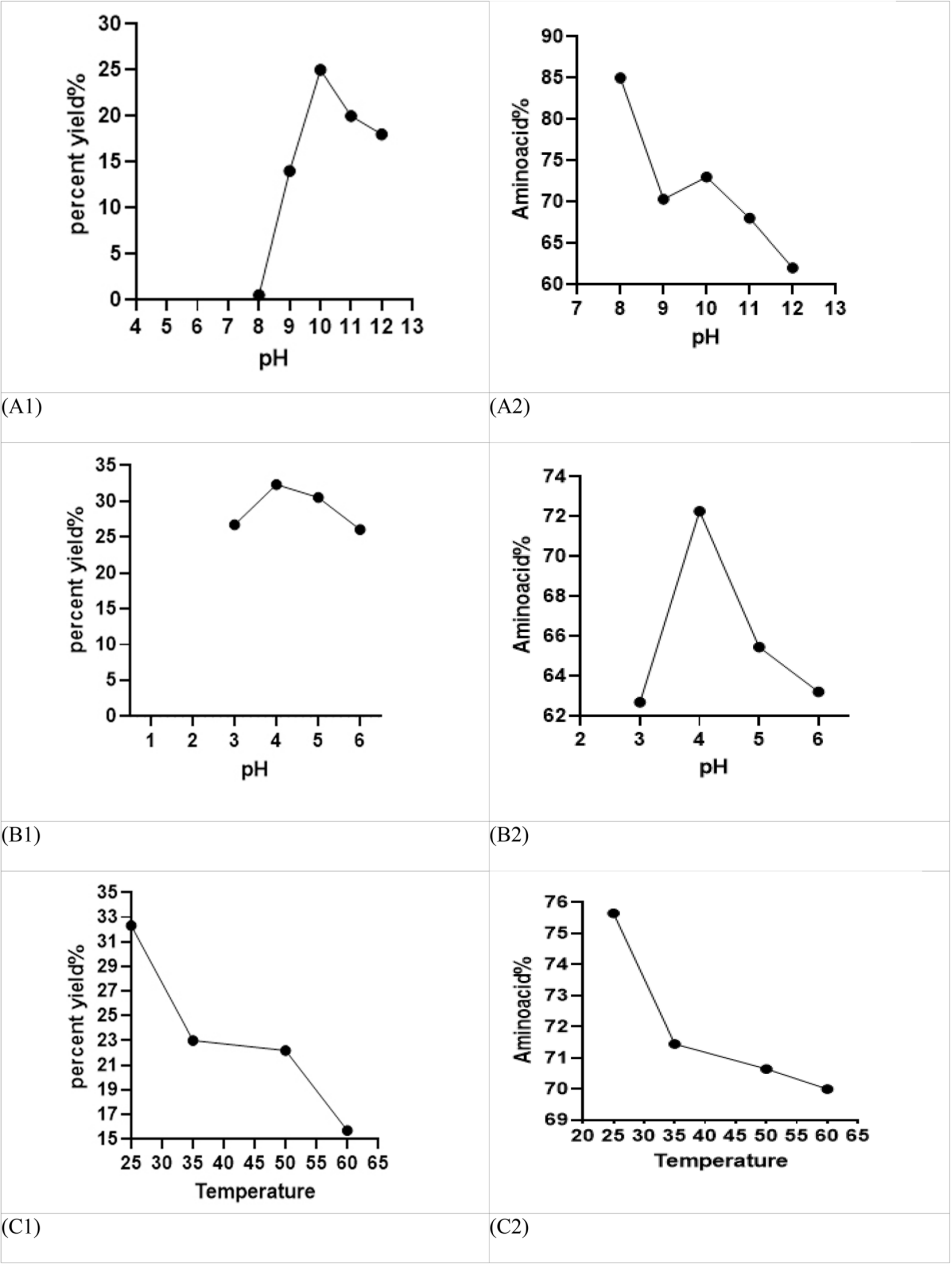
Percent yield and amino acid SOM conditions in alkaline pH (A1 and A2), percent yield and amino acid SOM conditions in acidic pH (B1 and B2), and percent yield (C1) and amino acid (C2) SOM conditions at different temperatures.

The results of different alkaline pH conditions on protein isolate percentage efficiency and total amino acid percentage in terms of l-alanine by the ninhydrin test in alkaline are presented in Fig. 3A1 and A2. The pH levels were 8.00, 9.00, 10.00, 11.00, and 12.00. Additionally, an acidic pH of equal to 5.00 at 25 °C was investigated. At pH 8.00, the amount of protein is higher than the other tests, but the efficiency percentage is very low. At pH 10.00, the amount of amino acid and its percentage efficiency are more suitable and affordable than the alkaline pH of 9.00–11.00–12.00. It was concluded that the lowest solubility can be found at an alkaline pH of 10.00, where the positive and negative charges are equal, and it is electrically neutral and completely precipitates; however, proteins have the lowest solubility at this point.^35^

### 3.4. Precipitation of proteins in acidic pH SOM

According to Fig. 3B1 and B2, the best neutralization pH, which is suitable in terms of efficiency and amino acid percentage, is pH 4.00. At pH lower than the isoelectric point, owing to the positive charge of the protein, the particles move away from each other with repulsive force, and at pH higher than the isoelectric point, due to the negative charge of the protein, they move away from each other and remain in solution. Therefore, at pH 4.00, most protein isolates is formed.

### 3.5. Investigating the total amino acid percentage of SOM at different temperatures

Temperature is an important factor in measuring the amount of protein isolation; the maximum amount of protein can be extracted at ambient temperature, and in terms of energy consumption, it is more economical. According to Fig. 3C1 and C2, it can be concluded that the efficiency of percentage and amount of amino acid at 25 °C is better compared to temperatures of 35 °C, 50 °C and 60 °C. Since high temperature can cause protein denaturation, the alkaline solution can easily hydrolyze the protein, and the molecular weight decreases; the extraction temperature is 25 °C, which is the best protein yield rate.^36^

### 3.6. Investigating the extraction efficiency of adding NaCl to SOM

The percentage of total amino acids increased by about 4.4% in the presence of salt; consequently, a higher efficiency percentage was obtained. The presence of lateral ionized branches in the protein causes changes in solubility in the presence of salt. The interaction between salt ions and some charged groups of protein is one of the important reasons for increasing protein solubility in salt solutions. By increasing the ionic strength of the salt solution, the degree of protein dissolution in the source material increases initially until it reaches its maximum value.^37,38^

### 3.7. Investigating protein of SOM, HOM, AOM and POM

The optimum conditions for sesame protein isolates are an alkaline pH of 10.00 and a neutralization pH of 4.00 at 25 °C for other protein isolates. Using the method of ninhydrin, the yield percentage and its amino acid were measured by a UV-Vis device at a wavelength of 570 nm. The results for hazelnut, almond and pumpkin were examined, as illustrated in Table 3. In this comparison, the amino acid percentage of almond, pumpkin, sesame, and hazelnut was the maximum in order, and by adding salt to the degreasing powder of seeds at 25 °C, the percentage efficiency increased.

**Table 3:** Investigation of the protein isolate of oilseed SOM, HOM, AOM and POM of oilseeds (95% confidence interval)

Row		pH alkaline	pH acidic	Temperature (°C)	Percentage yield%	Amino acid%
1	Sesame	10.00 ± 0.10	4.00 ± 0.13	25 ± 0.50	32.32 ± 0.11	75.60 ± 1.11
2	Hazelnut	10.00 ± 0.12	4.00 ± 0.15	25 ± 0.41	22.098 ± 0.10	76.00 ± 1.13
3	Almond	10.00 ± 0.11	4.00 ± 0.10	25 ± 0.52	25.12 ± 0.15	78.21 ± 0.99
4	Pumpkin	10.00 ± 0.10	4.00 ± 0.10	25 ± 0.55	26.83 ± 0.13	77.673 ± 1.14

### 3.8. Investigation of the amino acid profile of SOM, HOM, AOM and POM

A review of the amino acid profiles of SOM, HOM, AOM and POM is shown in Table 4. The results in Table 4 were obtained by HPLC. The abundance of amino acids in SOM and its protein percentages are very high. Consequently, it contains protein with an amino acid profile of good nutritional value. Considering the lack of animal protein sources, SOM can be a suitable substitute for daily protein consumption.^39,40^

**Table 4:** Investigation of the amino acid profile of SOM, HOM, AOM and POM (95% confidence interval)

Amino acids	SOM	HOM	AOM	POM
Glu	21.2 ± 0.11	16.60 ± 0.14	20.34 ± 0.12	14.643 ± 0.11
Gly	5.1 ± 0.13	3.09 ± 0.15	3.89 ± 0.15	3.86 ± 0.14
His	3.3 ± 0.15	1.02 ± 0.20	2.06 ± 0.14	1.88 ± 0.27
Arg	19.5 ± 0.11	10.30 ± 0.24	3.06 ± 0.11	12.11 ± 0.24
Ala	5.2 ± 0.12	4.30 ± 0.11	4.19 ± 0.16	4.5 ± 0.18
Tyr	2.9 ± 0.21	2.37 ± 0.16	2.58 ± 0.26	2.9 ± 0.19
Pro	3.1 ± 0.15	3.21 ± 0.18	7.35 ± 0.11	2.75 ± 0.11
Val	2.8 ± 0.23	3.42 ± 0.21	2.42 ± 0.14	4.6 ± 0.12
Ile	2.6 ± 0.18	2.95 ± 0.11	2.57 ± 0.19	2.08 ± 0.19
Lys	2.4 ± 0.14	2.45 ± 0.14	2.50 ± 0.21	3.15 ± 0.17
Leu	5.1 ± 0.12	5.97 ± 0.16	5.38 ± 0.14	6.59 ± 0.18
Phe	3.9 ± 0.19	4.08 ± 0.19	2.72 ± 0.15	2.38 ± 0.19
Met	2.6 ± 0.17	0.78 ± 0.16	0.64 ± 0.20	3.14 ± 0.23
ASP	5.87 ± 0.20	9.79 ± 0.21	12.54 ± 0.14	6.41 ± 0.13
Ser	4.9 ± 0.22	4.12 ± 0.23	3.6 ± 0.11	3.28 ± 0.24
Thr	6.8 ± 0.16	2.82 ± 0.17	1.72 ± 0.17	3.4 ± 0.14
Cys	0.9 ± 0.17	0.99 ± 0.15	0.65 ± 0.11	1.58 ± 0.16

The abundance of amino acids in HOM and its protein percentages are very high. The amino acid composition in hazelnut protein isolates is comparable to that of some animal proteins and is used as a suitable protein composition. According to Table 4, the HOM protein contains a high nutritional value. It can be considered an alternative and sustainable source for the production of vegetable protein, and owing to the growth of the world population, and ethical and environmental concerns, it can respond to the increasing demand for vegetable protein sources instead of animal protein.^16–41^

The abundance of amino acids in AOM and its protein percentages are very high. Owing to the appropriate protein percentage in almond protein isolates, it has a high nutritional value. According to Table 4, the production protein of AOM can be used to develop effective and environmentally friendly strategies for extracting the remaining oil and protein from AOM. It also leads to the production of high-quality protein and oil that can be used for food programs.^42^

The abundance of amino acids in the protein of POM and its protein percentage are very high. According to Table 4, the POM protein is a suitable source of vegetable protein and has a high nutritional value. Therefore, it can respond to the increasing demand for vegetable protein sources instead of animal protein.^19,20^

The oil extraction industry produces a lot of waste, and the disposal of these wastes is costly and problematic from economic and environmental perspectives. These wastes also have high nutritional and medicinal value, so they were used in various processes that led to the production of a product with high nutritional value, and the resulting production waste was minimized. The general use of oilseed meals means valuing their protein part in the production of value-added products for the food, cosmetic and pharmaceutical industries. Among the protein sources used in livestock and poultry diets, oilseed meals are very important. The production of protein isolates of seeds for breeding and the rest of the animal generation as an effective feed is of great importance to the eater. Changing lifestyles in developed and developing countries have affected the eating habits of consumers and encouraged the use of ready-made and low-volume foods, and public awareness about the benefits of eating plant-based protein products, such as low-calorie and fiber-containing products. There is an increasing trend of avoiding animal fats. Common sources of protein include red meat, chicken, eggs, dairy products and fish.^43^

The rapid growth of the population, the limitation of food sources and the increase in demand for new and cheap protein sources with favorable functional characteristics have directed the attention of scientists to plant protein sources, especially oilseeds, rice bran, alfalfa, peas, walnuts, *etc.* Research investments in agriculture and agri-food technology should be considered to develop the production of protein-rich seeds, valorize previously consumed plant protein fractions and develop a new generation of protein extracts from agricultural sources, such as oilseed meal, for human nutrition.^11^

Several studies have been conducted on protein extraction methods, and alkaline extraction and isoelectric point precipitation are the most common methods for preparing protein isolates in the food industry.^9–11^

## 4. Conclusion

In this study, the alkaline extraction method for the preparation of sesame, hazelnut, almond and pumpkin protein isolate was investigated. The amounts of protein in the protein isolates containing sesame, hazelnut, almond and pumpkin obtained using the ninhydrin method were 76.20, 79.05, 81.04, and 80.22, respectively. Additionally, to test the validity of the protein content in an oilseed meal, the Kjeldahl method was used. To check the amino acid profile of an oilseed meal containing sesame, hazelnut, almond, and pumpkin, the HPLC technique was used. Favorable results were obtained, which show that it contains an abundance of amino acids in the protein isolate of oilseeds and its protein percentage is very high. The investigated temperature of 25 °C was chosen because the highest percentage of efficiency and the highest percentage of amino acid were obtained. In this investigation, the presence of NaCl salt at a rate of 0.1% presented favorable results. As the salt concentration increases, protein surface charges interact with salt instead of interacting with water. Consequently, the hydrophobic parts of the protein are placed on the surface, causing the proteins to stick together and precipitate.

## Conflicts of interest

There are no conflicts to declare.

## Data Availability

The data supporting this article have been included as part of the manuscript. For requests about the original data, please do not hesitate to contact the corresponding author.
